# MiRNA-Related SNPs and Risk of Esophageal Adenocarcinoma and Barrett’s Esophagus: Post Genome-Wide Association Analysis in the BEACON Consortium

**DOI:** 10.1371/journal.pone.0128617

**Published:** 2015-06-03

**Authors:** Matthew F. Buas, Lynn Onstad, David M. Levine, Harvey A. Risch, Wong-Ho Chow, Geoffrey Liu, Rebecca C. Fitzgerald, Leslie Bernstein, Weimin Ye, Nigel C. Bird, Yvonne Romero, Alan G. Casson, Douglas A. Corley, Nicholas J. Shaheen, Anna H. Wu, Marilie D. Gammon, Brian J. Reid, Laura J. Hardie, Ulrike Peters, David C. Whiteman, Thomas L. Vaughan

**Affiliations:** 1 Division of Public Health Sciences, Fred Hutchinson Cancer Research Center, Seattle, Washington, United States of America; 2 Department of Biostatistics, University of Washington, School of Public Health, Seattle, Washington, United States of America; 3 Department of Chronic Disease Epidemiology, Yale School of Public Health, New Haven, CT, United States of America; 4 Department of Epidemiology, MD Anderson Cancer Center, Houston, TX, United States of America; 5 Pharmacogenomic Epidemiology, Ontario Cancer Institute, Toronto, Ontario, Canada, M5G 2M9; 6 Medical Research Council (MRC) Cancer Cell Unit, Hutchison-MRC Research Centre and University of Cambridge, Cambridge, United Kingdom; 7 Department of Populations Sciences, Beckman Research Institute and City of Hope Comprehensive Cancer Center, Duarte, California, United States of America; 8 Department of Medical Epidemiology and Biostatistics, Karolinska Institutet, Stockholm, Sweden; 9 Department of Oncology, Medical School, University of Sheffield, Sheffield, United Kingdom; 10 Division of Gastroenterology and Hepatology, Mayo Clinic, Rochester, Minnesota, United States of America; 11 The Romero Registry, Mayo Clinic, Rochester, Minnesota, United States of America; 12 Department of Surgery, University of Saskatchewan, Saskatoon, SK, Canada; 13 Division of Research, Kaiser Permanente Northern California, Oakland, California, United States of America; 14 San Francisco Medical Center, Kaiser Permanente Northern California, San Francisco, California, United States of America; 15 Division of Gastroenterology and Hepatology, University of North Carolina School of Medicine, University of North Carolina, Chapel Hill, North Carolina, United States of America; 16 Department of Preventive Medicine, University of Southern California/Norris Comprehensive Cancer Center, Los Angeles, California, United States of America; 17 Department of Epidemiology, University of North Carolina School of Public Health, Chapel Hill, North Carolina, United States of America; 18 Division of Human Biology, Fred Hutchinson Cancer Research Center, Seattle, Washington, United States of America; 19 Division of Epidemiology, University of Leeds, Leeds, United Kingdom; 20 Department of Epidemiology, University of Washington, School of Public Health, Seattle, Washington, United States of America; 21 Cancer Control, QIMR Berghofer Medical Research Institute, Brisbane, Australia; MOE Key Laboratory of Environment and Health, School of Public Health, Tongji Medical College, Huazhong University of Science and Technology, CHINA

## Abstract

Incidence of esophageal adenocarcinoma (EA) has increased substantially in recent decades. Multiple risk factors have been identified for EA and its precursor, Barrett’s esophagus (BE), such as reflux, European ancestry, male sex, obesity, and tobacco smoking, and several germline genetic variants were recently associated with disease risk. Using data from the Barrett’s and Esophageal Adenocarcinoma Consortium (BEACON) genome-wide association study (GWAS) of 2,515 EA cases, 3,295 BE cases, and 3,207 controls, we examined single nucleotide polymorphisms (SNPs) that potentially affect the biogenesis or biological activity of microRNAs (miRNAs), small non-coding RNAs implicated in post-transcriptional gene regulation, and deregulated in many cancers, including EA. Polymorphisms in three classes of genes were examined for association with risk of EA or BE: miRNA biogenesis genes (157 SNPs, 21 genes); miRNA gene loci (234 SNPs, 210 genes); and miRNA-targeted mRNAs (177 SNPs, 158 genes). Nominal associations (P<0.05) of 29 SNPs with EA risk, and 25 SNPs with BE risk, were observed. None remained significant after correction for multiple comparisons (FDR q>0.50), and we did not find evidence for interactions between variants analyzed and two risk factors for EA/BE (smoking and obesity). This analysis provides the most extensive assessment to date of miRNA-related SNPs in relation to risk of EA and BE. While common genetic variants within components of the miRNA biogenesis core pathway appear unlikely to modulate susceptibility to EA or BE, further studies may be warranted to examine potential associations between unassessed variants in miRNA genes and targets with disease risk.

## Introduction

Incidence of esophageal adenocarcinoma (EA) in Western countries has risen sharply in recent decades, while median survival remains less than one year [1]. EA typically arises within a columnar metaplastic precursor epithelium known as Barrett’s esophagus (BE). Established risk factors for EA and BE include symptomatic gastroesophageal reflux disease (GERD), European ancestry, male sex, obesity, and tobacco smoking [2]. Less is known about the role of inherited genetic variation and its interplay with environmental factors. Candidate-gene-based studies have associated altered risk of EA or BE with DNA polymorphisms in genes that function in a wide range of biological pathways (inflammation, detoxification, DNA repair, angiogenesis, and apoptosis) [3–13], while a recent linkage-based genetic analysis of sibling pairs provided preliminary evidence for several novel germline mutations [14]. These studies have been limited by small sample sizes and the need for validation. In the last few years, large genome-wide association studies of BE and EA identified multiple SNPs significantly associated with disease risk [15–18], including variants located in three transcription factors, a transcriptional co-activator, and the major histocompatibility complex locus, none of which were previously implicated by candidate-based studies.

A large body of work has established that microRNAs (miRNAs), small non-coding RNAs that function in post-transcriptional gene regulation, act as oncogenes or tumor suppressors in a variety of tissues, and their deregulation can lead to cancer [19,20]. MiRNA gene loci are transcribed by RNA polymerase II to generate primary (pri-) miRNA transcripts, which are then cleaved by the nuclear RNAse DROSHA complex to form stem-loop precursor (pre-) miRNAs. Following nuclear export to the cytoplasm, pre-miRNAs undergo further processing by the DICER complex to generate mature miRNAs, which typically bind to the 3’ untranslated region of target messenger RNAs (mRNAs) and mediate translational repression or RNA degradation. MiRNAs have been linked to inflammatory pathways [21,22], which are likely to play important roles in BE/EA. Changes in miRNA expression have been detected at multiple stages in the development of EA [23–38], and certain miRNAs may be associated with prognosis [33,35].

Many studies have reported associations between miRNA-related SNPs and risk of diverse cancers [39]. These SNPs may reside in a) miRNA biogenesis genes, b) miRNA gene loci, or c) miRNA-targeted mRNAs. Functional miRNA-related SNPs may affect global miRNA expression levels, processing or expression of individual miRNAs, and miRNA target gene specificity. A previous case-control study of 346 esophageal cancer cases (86% EA) and an equal number of matched controls reported that seven miRNA-related SNPs from a panel of 41 total SNPs tested were associated with altered risk of esophageal carcinoma, with the association of one SNP in the pre-miRNA-423 region remaining statistically significant after correction for multiple comparisons [40]. These SNPs have not been validated in independent study populations or evaluated for potential associations with BE, and it is currently unknown whether additional SNPs in the miRNA pathway may modulate disease risk. Using data from a recent genome-wide association study (GWAS) of 2,515 EA cases, 3,295 BE cases, and 3207 controls [16], we selected 157 biogenesis pathway SNPs, 234 miRNA SNPs, and 177 mRNA target SNPs and assessed their associations with risks of EA and BE.

## Methods

### Study population and SNP genotyping

The Barrett’s and Esophageal Adenocarcinoma Genetic Susceptibility Study (BEAGESS) included men and women diagnosed with EA or BE, and control participants pooled from 14 individual studies conducted in Western Europe, Australia, and North America over the past twenty years. Detailed study population characteristics and genotyping protocols have been published [16]. Briefly, all EA and BE case participants were confirmed by histologic examination, and a set of population control individuals was drawn from the included Barrett’s and Esophageal Adenocarcinoma Consortium (BEACON) studies to serve as a comparison group for both EA and BE case participants. The current analysis employed a pooled dataset that has been described previously [41], and included all BEAGESS participants, additional BE and EA patients from the UK Barrett’s Esophagus Gene Study and the UK Stomach and Oesophageal Cancer Study (SOCS), respectively [16], and additional controls from a hospital-based case-control study of melanoma conducted at the MD Anderson Cancer Center (Houston, TX) [42]. Genotyping of buffy coat or whole blood DNA from all participants was conducted using the Illumina Omni1M Quad platform, in accordance with standard quality control procedures [43]. All participants gave written informed consent, and this project was approved by the ethics review board of the Fred Hutchinson Cancer Research Center. We selected all unrelated participants with missing genotyping call rates < 2%; thus the final study sample included 2,515 EA cases, 3,295 BE cases, and 3,207 controls. Three control participants were excluded from analyses involving BE cases, because of familial relation to cases.

### Selection of miRNA-related SNPs

SNPs selected for this study are located in or near a) miRNA biogenesis genes (+/- 2kb), b) miRNA gene loci (+/- 25bp), or c) predicted or verified mRNA targets of miRNAs (**S1 Fig**). We excluded from consideration SNPs that failed Illumina quality measures or standard quality control procedures [43]. Specifically, SNPs were excluded if any of the following criteria were satisfied: i) Illumina GenTrain score < 0.6 or cluster separation < 0.4; ii) >5% missing call rate over samples; iii) discordant genotype calls in any pair of duplicate study samples; iv) Mendelian error in either one of the HapMap QC trios or the small number of families identified in the BEACON data; v) significant departure from Hardy-Weinberg Equilibrium (P<10^–4^); vi) minor allele frequency (MAF) < 1%.

After imposing the above filters, we first identified all available Omni1M SNPs (n = 185) located within fourteen genes in the miRNA biogenesis core pathway (2kb flanking sequences for each gene were also included, using gene boundaries defined in hg19/GRChB37): *DDX20*, *DGCR8*, *DICER1*, *DROSHA*, *EIF2C1-4*, *GEMIN4*, *GW182*, *PRKRA*, *RAN*, *TARBP2*, *XPO5*. Similarly, we identified all available Omni1M SNPs (n = 70) located within 1601 human miRNA precursor sequences (+/- 25bp) deposited in miRBase (version 19). Additional Omni1M SNPs (n = 160) in pairwise linkage disequilibrium (LD) (*r* ^*2*^>0.8) with non-Omni1M dbSNP polymorphisms located within the 1601 miRNA precursors (+/-25bp) were also identified, using 1000 Genomes data for the CEU population via the SNP Annotation and Proxy (SNAP) website (http://www.broadinstitute.org/mpg/snap). In the case of multiple possible Omni1M proxy SNPs, selection was based on maximum *r* ^*2*^ coefficient followed by minimum distance (bp). Within these two categories of SNPs (biogenesis and miRNA gene loci), LD-based pruning was carried out with pairwise LD data from SNAP, using an *r* ^*2*^ threshold of 0.8, to reduce the number of redundant tests (126/185 biogenesis SNPs and 229/230 miRNA gene SNPs were retained).

To identify potentially functional SNPs in miRNA-targeted mRNAs, we used a database of polymorphisms predicted to alter miRNA-mRNA regulation [44]. Two filters were imposed to limit the set of SNPs to those most likely to be functional. First, we considered miRNA-mRNA interactions only for miRNAs shown to be expressed in the esophagus at some point in the disease progression from normal squamous epithelium to BE/EA. Based on the union of several published reports [32,34–38], 135 expressed miRNAs were identified (**S1 Table**). Second, after the previous exclusions, we considered only a subset of miRNA-mRNA interactions (≈3%) that were predicted to be most strongly affected by genetic variants in the target mRNA (ΔS>0.85, where S is the predicted regulation score for a given miRNA:mRNA pairing). LD-based pruning of these filtered SNPs (n = 175) was performed as described previously (145/175 SNPs were retained).

A literature search was also conducted to identify miRNA-related SNPs shown to be associated with cancer susceptibility (**S2 Table**) [39,45–67]. Variants not already captured by our selection process were added if available in the Omni1M dataset, or proxy SNPs in high LD were substituted where possible, as indicated (**S3 Table**). The final set of polymorphisms included 157 biogenesis pathway SNPs (21 genes), 234 miRNA SNPs (210 genes), and 177 target mRNA SNPs (158 genes). Minor and major alleles were reported throughout using the ‘plus’ strand designation.

### Statistical analysis

Unconditional multivariate logistic regression was used to compute odds ratios for risk of EA or BE associated with a given SNP variant, using an additive model (per-allele), while adjusting for age, sex, and the first four principal components (PCs) derived from principal components analysis (PCA) to account for population stratification by ancestry [41]. Inclusion of the first four PCs was empirical, as described previously in the parent GWAS report [16], and based on assessment of i) the percentage of variance accounted for (**S2 Fig**) and ii) the observed separation of study participants according to pairwise comparisons of individual principal components (**S3 Fig**). To correct for multiple comparisons when assessing statistical significance, we used the Bonferroni or Benjamini-Hochberg false-discovery rate (FDR) methods [68,69]. The threshold for Bonferroni significance was α = 0.05/568 = 8.80x10^-5^. Stratified analyses by body mass index (BMI, kg/m^2^) and history of cigarette smoking were also conducted, and evidence for interaction was assessed by including a product term in the logistic regression model. Smoking history and BMI were defined categorically in stratified analyses (smoking: ever/never, or pack-years: 0, >0 & <15, 15–29, 30–44, 45+; BMI: <25, 25–29, 30–34, 35+), and continuously (pack-years, BMI) to test for interaction. Statistical analyses were conducted using STATA/SE version 13.1 (College Station, TX).

## Results

### Characteristics of study participants

The distributions of demographic characteristics among controls, EA cases, and BE cases are shown in **Table 1**. EA cases were somewhat older (mean age: 65.0 years) and more frequently male (87%) relative to controls (mean age: 58.4 years, 73% male) and BE cases (mean age: 63.0 years, 76% male). Among participants with non-missing data for BMI and smoking history, the percentage reporting ever having smoked cigarettes was higher among EA (75%) and BE (66%) cases than among controls (59%). Heavy smoking (45+ pack years) was more prevalent among EA cases (21%) than among controls (14%) or BE cases (14%), while obesity (BMI 30+) was more prevalent among EA (30%) and BE (37%) cases than among controls (20%).

**Table 1:** Characteristics of controls, EA cases, and BE cases.

	Controls		EA		BE	
	(n = 3207)		(n = 2515)		(n = 3295)	
	n	%	n	%	n	%
Age^a^						
<50	726	22.6	189	7.6	449	13.7
50–59	885	27.6	547	21.9	780	23.7
60–69	963	30.0	884	35.4	1011	30.7
70+	633	19.7	875	35.1	1048	31.9
Sex						
Female	880	27.4	320	12.7	806	24.5
Male	2327	72.6	2195	87.3	2489	75.5
BMI^a^						
<25	786	36.3	245	24.6	425	20.7
25–29.99	944	43.6	455	45.7	882	42.9
30–34.99	307	14.2	201	20.2	521	25.3
35+	130	6.0	95	9.5	230	11.2
Smoking status^a^						
no	889	40.9	348	24.7	798	33.7
yes	1284	59.1	1062	75.3	1570	66.3
Smoking (p-y)^a^ ^,^ ^b^						
none	889	41.3	348	32.8	798	44.5
<15	358	16.6	156	14.7	320	17.9
15–29	326	15.1	160	15.1	232	12.9
30–44	273	12.7	173	16.3	198	11.0
45+	309	14.3	225	21.2	244	13.6

^a^Numbers do not add to total subjects due to missing data

^b^pack-years

### Associations of miRNA-related SNPs and risk of EA or BE

Of the 157 biogenesis pathway SNPs, 234 miRNA SNPs, and 177 mRNA target SNPs evaluated in this study (**S3 Table**), 29 were nominally associated (P<0.05) with risk of EA (**Table 2 and S4 Fig**), and 25 with risk of BE (**Table 3**). Three SNPs satisfied P<0.05 in both analyses (EA and BE): *RDH8* rs1644730 T>A, *miR-3117* rs7526812 T>C, and *miR-4467* rs12534337 G>A. For each of these SNPs, the risk estimates for EA and BE were similar and in the same directions (OR 0.88 vs 0.93, 1.14 vs 1.11, 1.28 vs 1.25, respectively) (**S4 Table**). After correction for multiple comparisons (n = 568 total SNPs), using the Bonferroni or false discovery rate method, none of the observed associations remained statistically significant (FDR q>0.50).

**Table 2:** MiRNA-related SNPs and risk of EA.^#^

**Biogenesis pathway**											
				**Controls**		**EA cases**					
	**SNP**	**Gene**	**Alleles†**	**N**	**MAF** ^**‡**^	**N**	**MAF** ^**‡**^	OR*	**95% CI**	**P**	**q**
**1**	kgp1460594	XPO5	A/G	3207	0.075	2495	0.091	1.21	(1.05–1.40)	0.0074	0.54
**2**	rs7702984	DROSHA	G/A	3206	0.012	2494	0.009	0.63	(0.43–0.94)	0.0227	0.70
**3**	rs4351606	EIF2C3	A/G	3205	0.056	2493	0.047	0.82	(0.69–0.98)	0.0327	0.84
**miRNA genes**											
				**Controls**		**EA cases**					
	**SNP**	**Gene**	**Alleles†**	**N**	**MAF** ^**‡**^	**N**	**MAF** ^**‡**^	OR*	**95% CI**	**P**	**q**
**1**	rs17880825	miR-4725	C/T	3206	0.020	2494	0.028	1.46	(1.13–1.89)	0.0037	0.54
**2**	rs1709696	miR-3612	A/C	3205	0.227	2493	0.208	0.88	(0.80–0.97)	0.0086	0.54
**3**	rs1378940	miR-4513	C/A	3206	0.343	2495	0.314	0.89	(0.82–0.97)	0.0091	0.54
**4**	rs12534337	miR-4467	A/G	3205	0.042	2495	0.053	1.28	(1.06–1.55)	0.0095	0.54
**5**	rs12564376	miR-4421	A/G	3207	0.039	2495	0.030	0.75	(0.60–0.94)	0.0108	0.55
**6**	rs7526812	miR-3117	C/T	3207	0.151	2495	0.165	1.14	(1.02–1.27)	0.0195	0.70
**7**	rs7210250	miR-4521	G/A	3206	0.019	2493	0.015	0.70	(0.51–0.94)	0.0201	0.70
**8**	rs7342570	miR-487a	T/C	3206	0.197	2495	0.213	1.12	(1.02–1.24)	0.0204	0.70
**9**	rs3787547	miR-4756	A/G	3205	0.435	2495	0.415	0.91	(0.84–0.99)	0.0236	0.70
**10**	rs9842591	miR-5186	A/C	3207	0.459	2494	0.475	1.09	(1.01–1.18)	0.0280	0.78
**11**	rs12232826	miR-638	T/G	3207	0.047	2495	0.035	0.81	(0.66–0.99)	0.0356	0.86
**12**	rs12461701	miR-3188	A/G	3206	0.278	2495	0.259	0.91	(0.83–0.99)	0.0366	0.86
**13**	rs10899620	miR-5579	C/T	3199	0.194	2490	0.185	0.90	(0.82–1.00)	0.0435	0.87
**mRNA targets**											
				**Controls**		**EA cases**					
	**SNP**	**Gene**	**Alleles†**	**N**	**MAF** ^**‡**^	**N**	**MAF** ^**‡**^	OR*	**95% CI**	**P**	**q**
**1**	rs1644730	RDH8	A/T	3202	0.472	2495	0.441	0.88	(0.81–0.95)	0.0011	0.54
**2**	rs1045968	PRRT2	A/C	3206	0.141	2494	0.160	1.17	(1.05–1.31)	0.0046	0.54
**3**	rs1050629	MLF2	A/G	3206	0.025	2495	0.033	1.40	(1.10–1.78)	0.0055	0.54
**4**	rs2075993	E2F2	T/C	3206	0.499	2494	0.480	0.90	(0.83–0.97)	0.0072	0.54
**5**	rs9804386	MORN4	C/T	3207	0.200	2495	0.221	1.14	(1.03–1.25)	0.0088	0.54
**6**	rs1799782	XRCC1	T/C	3207	0.066	2495	0.053	0.82	(0.69–0.97)	0.0207	0.70
**7**	rs1367	SCUBE2	G/A	3207	0.075	2494	0.063	0.83	(0.71–0.97)	0.0225	0.70
**8**	rs11169571	ATF1	C/T	3206	0.391	2494	0.424	1.10	(1.01–1.19)	0.0237	0.70
**9**	rs2521795	GDE1	A/G	3206	0.151	2495	0.162	1.13	(1.01–1.26)	0.0289	0.78
**10**	rs3209160	ZDHHC21	C/G	3201	0.137	2490	0.153	1.13	(1.01–1.26)	0.0384	0.87
**11**	rs3660	KRT81	G/C	3198	0.470	2490	0.485	1.09	(1.00–1.17)	0.0414	0.87
**12**	rs3174352	ASPN	A/G	3206	0.504	2493	0.482	0.92	(0.86–1.00)	0.0493	0.87
**13**	rs11550670	STXBP6	T/C	3204	0.204	2491	0.220	1.10	(1.00–1.22)	0.0495	0.87

^#^nominal P<0.05

^†^Minor/major alleles

^**‡**^Minor allele frequency

*OR adjusted for age, sex, PC1-PC4, using additive model (per-allele)

**Table 3:** MiRNA-related SNPs and risk of BE.^#^

**Biogenesis pathway**											
				**Controls**			**BE cases**				
	**SNP**	**Gene**	**Alleles†**	**N**	**MAF** ^**‡**^	**N**	**MAF** ^**‡**^	OR*	**95% CI**	**P**	**q**
**1**	rs8192593	TARBP2	T/C	3201	0.040	3286	0.034	0.77	(0.63–0.93)	0.0063	0.89
**2**	rs595055	EIF2C1	C/T	3202	0.142	3288	0.125	0.88	(0.79–0.97)	0.0136	0.89
**3**	rs2944760	EIF2C2	C/A	3198	0.199	3287	0.186	0.90	(0.82–0.98)	0.0198	0.89
**4**	rs12741800	LIN28	A/G	3198	0.458	3276	0.442	0.92	(0.86–0.99)	0.0231	0.89
**5**	rs11247946	LIN28	G/A	3204	0.345	3288	0.327	0.92	(0.85–0.99)	0.0295	0.89
**6**	rs538779	DDX20	A/G	3204	0.217	3288	0.202	0.91	(0.83–0.99)	0.0323	0.89
**7**	rs6598964	LIN28	T/C	3204	0.368	3288	0.348	0.93	(0.86–1.00)	0.0391	0.89
**8**	rs12044203	EIF2C4	A/G	3194	0.021	3284	0.014	0.75	(0.57–0.99)	0.0420	0.89
**miRNA genes**											
				**Controls**		**BE cases**					
	**SNP**	**Gene**	**Alleles†**	**N**	**MAF** ^**‡**^	**N**	**MAF** ^**‡**^	OR*	**95% CI**	**P**	**q**
**1**	rs3785722	miR-1269b	A/G	3203	0.449	3288	0.422	0.89	(0.83–0.96)	0.0021	0.89
**2**	rs10906086	miR-548ak	C/A	3203	0.469	3286	0.490	1.10	(1.03–1.19)	0.007	0.89
**3**	rs7000768	miR-3686	G/A	3204	0.304	3288	0.325	1.11	(1.02–1.19)	0.0104	0.89
**4**	rs12534337	miR-4467	A/G	3202	0.042	3287	0.052	1.25	(1.05–1.48)	0.0106	0.89
**5**	rs10862193	miR-617	G/A	3200	0.428	3281	0.409	0.92	(0.86–0.99)	0.0206	0.89
**6**	rs4369899	miR-4431	C/T	3204	0.338	3285	0.316	0.91	(0.85–0.99)	0.0214	0.89
**7**	rs2114358	miR-1206	G/A	3204	0.391	3288	0.408	1.09	(1.01–1.17)	0.0273	0.89
**8**	rs17252270	miR-548x-2	T/C	3203	0.140	3286	0.150	1.12	(1.01–1.24)	0.0283	0.89
**9**	rs7211449	miR-548h-3	A/C	3203	0.211	3288	0.196	0.91	(0.83–0.99)	0.0347	0.89
**10**	rs10840491	miR-4686	T/C	3203	0.139	3288	0.125	0.90	(0.81–1.00)	0.0447	0.89
**11**	rs7526812	miR-3117	C/T	3204	0.151	3287	0.159	1.11	(1.00–1.22)	0.0456	0.89
**mRNA targets**											
				**Controls**		**BE cases**					
	**SNP**	**Gene**	**Alleles†**	**N**	**MAF** ^**‡**^	**N**	**MAF** ^**‡**^	OR*	**95% CI**	**P**	**q**
**1**	rs1043681	THAP3	C/T	3204	0.316	3288	0.301	0.90	(0.83–0.97)	0.0064	0.89
**2**	rs7248876	ZNF181	G/A	3200	0.333	3286	0.357	1.10	(1.02–1.18)	0.0139	0.89
**3**	rs1043420	CAPN5	C/T	3203	0.222	3288	0.236	1.10	(1.01–1.20)	0.0247	0.89
**4**	rs13043447	CABLES2	T/C	3204	0.080	3287	0.071	0.86	(0.76–0.99)	0.0324	0.89
**5**	rs1043641	ACBD3	A/G	3204	0.172	3288	0.158	0.90	(0.82–0.99)	0.0331	0.89
**6**	rs1644730	RDH8	A/T	3199	0.473	3285	0.453	0.93	(0.86–1.00)	0.0384	0.89

^#^nominal P<0.05

^†^Minor/major alleles

^**‡**^Minor allele frequency

*OR adjusted for sex, PC1-PC4, using additive model (per-allele)

### Stratified analyses by smoking history and BMI

Stratified analyses were conducted to determine whether or not selected top candidates listed in Tables 2 & 3 (P<7.5x10^-3^) exhibited disease-risk associations that varied by pack-years of smoking history or by BMI (**S5 and S6 Tables**). Assessment of all 568 variants revealed multiple nominally-significant interactions with smoking history or BMI, but none remained significant after correction for multiple comparisons (FDR q>0.05). One variant (rs595055 T>C) in the biogenesis gene *EIF2C1* exhibited a borderline-significant interaction (P = 0.0001, q = 0.057) with BMI in relation to risk of BE, with elevated disease risk seen only among obese individuals.

## Discussion

Using genotyping data from a recent consortium-based GWAS, we evaluated the association of 568 miRNA-related SNPs with risks of EA and BE. In total, 29 SNPs were found to be nominally associated (P<0.05) with risk of EA, and 25 with risk of BE, with three shared variants identified between these analyses. None remained significant after correction for multiple comparisons.

Aberrant expression of miRNAs has been reported in many cancers, and several studies have described miRNA expression changes at specific stages in the development of EA, which may be associated with progression or prognosis [24,29,31,33,35,36]. Inherited genetic variation in the miRNA pathway has been linked to altered susceptibility to a variety of cancers, but few studies have focused on esophageal cancer, and in particular, EA (as opposed to esophageal squamous cell carcinoma, ESCC) [39,40,70,71]. The largest previous study included 386 esophageal cancer cases (86% EA, n = 296) [40], and identified seven SNPs significantly associated with cancer risk, five of which were associated specifically with EA. Of those, a single SNP in the pre-miR-423 region remained significant after adjustment for multiple comparisons. This variant failed to validate in our own present study of >2,500 EA cases and >3,200 controls.

Since BE is an established risk factor for, and the only known precursor of, EA, it was of interest to compare the list of SNPs nominally associated with risk of each condition. For each of the three variants reaching P<0.05 in both analyses (*RDH8* rs1644730 T>A, *miR-3117* rs7526812 T>C, and *miR-4467* rs12534337 G>A), the direction and magnitude of the OR was very similar for BE and EA (**S4 Table**), raising the possibility that the association with risk of BE might account for the association with risk of EA. However, given our use of a single control group for comparison to both the EA and BE case groups, cautious interpretation is required, since some shared associations could be expected by chance alone. When EA and BE cases were combined into a single case group and compared to the same set of controls, all three of these variants were highly-ranked among the set of 31 nominally significant SNPs (**S7 Table**), and two of the three (rs7526812 T>C, rs12534337 G>A) had smaller P values than observed in the individual analyses, without reaching Bonferroni or FDR significance thresholds.

rs1644730 T>A is located within a predicted miR-630 binding site in the 3’UTR of all-trans-retinol dehydrogenase 8 (*RDH8*), which encodes a short-chain dehydrogenase/reductase enzyme involved in rhodopsin regeneration in the vision pathway. Of interest, the enzymatic activity of RDH8 has also been linked to estrogen biosynthesis [72]. Given the significantly higher incidence of BE/EA among males versus females, a potential protective effect of estrogens has been proposed, and some evidence exists for reduced disease risk associated with hormone replacement therapy [73]. An intriguing hypothesis relates to whether the suggested inverse association observed in this study for rs1644730 T>A may reflect impaired miRNA-mediated repression of RDH8 and consequent elevations in estrogen synthesis. In exploratory analyses stratified by sex, the inverse association of this variant with risk of EA appeared stronger among males (OR = 0.86, p = 0.00057, q = 0.23) relative to the overall study sample (OR = 0.88, p = 0.0011, q = 0.54), and was not evident among the limited pool (n = 1200) of female participants (OR = 0.97, p = 0.79). rs12534337 G>A is located in the miR-4467 precursor, identified by deep sequencing of the small RNA transcriptome of normal and malignant B cells, and paired normal and tumor breast tissue (68, 69). Expression of miR-4467 has not been assessed in the esophagus, and no studies have examined functional effects of this miRNA. The proto-oncogene *JUNB* mRNA is predicted by the TargetScan algorithm to be one of 23 conserved targets of miR-4467, but experimental validation has not been reported. Any influence of this SNP on the biogenesis of miR-4467 remains to be determined. rs7526812 T>C is located ~15kb downstream from, and is in strong linkage disequilibrium (*r* ^*2*^ = 1.0) with, a variant in the miR-3117 gene locus (rs12402181). rs7526812 T>C is also a missense polymorphism situated within an exon of the *SGIP* gene, which has been genetically linked to fat mass [74].

Five of the seven SNPs reported to be associated with risk of esophageal cancer (EA/ESCC) by Ye *et al*. [40] were included in our analysis of genotyped SNPs from the BEACON GWAS, including their single SNP (pre-miR-423 rs6505162) reported as significant after correction for multiple comparisons. Three of these five SNPs had been associated with EA (*miR-423* rs6505162, *miR-196a-2* rs11614913, and *RAN* rs14035), while two reached borderline significance (*XPO5* rs11077 and pri-miR-219-1 rs213210). In our study, none of these five SNPs were (nominally) associated (P<0.05) with either EA or BE (**S8 Table**), and nearly all ORs were very close to 1. Ye *et al*. evaluated three genetic models (additive, recessive, dominant) and reported the best-fitting model in their analysis, in contrast to our approach of assessing exclusively the additive model. Re-analysis of these five SNPs using the alternative models resulted in one polymorphism reaching borderline significance (pri-miR-219-1 rs213210 T>C, dominant model, EA OR 1.17, 95% CI 1.01–1.47, P = 0.06), in the same direction as the previously published results (OR 1.61, P = 0.058). Ye *et al*. did not evaluate any of the nominally-associated variants identified in our analysis. Multiple factors could account for discrepancies between studies. First, many apparent signals in association studies may be false positives, and replication in large, independent populations is critical (our EA analysis included more than eight times as many cases as the previous study). Second, different approaches were taken in adjustment for covariates. While both studies included only individuals of European ancestry, we also adjusted for population stratification via inclusion of several PCs derived from principal components analysis, but chose not to adjust for smoking status.

Strengths of our study included the use of pooled data from the BEACON GWAS, which provided the largest sample size to date in the evaluation of miRNA-related SNPs and risks of EA and BE. Inclusion of both BE and EA cases allowed for a comparison of the genetic variation associated with risk of a neoplastic precursor lesion and the cancer that arises from it. The availability of covariate data for smoking history and BMI further enabled us to evaluate gene-environment interactions for two established risk factors for these conditions. Our assessment of 568 polymorphisms significantly expands upon past efforts to examine genetic variation in miRNA pathways in relation to risk of Barrett’s esophagus and esophageal adenocarcinoma. In particular, our inclusion of >120 SNPs within 14 genes implicated in miRNA biogenesis allowed for broad coverage of this canonical core pathway, activity of which underlies the global expression of miRNAs.

This study also had certain limitations. First, while the EA/BE GWAS employed in our analyses represents the largest study of its kind currently available, it remains plausible that among the variants examined, some true associations of small magnitude were missed due to power constraints, especially those present exclusively in population sub-groups. Missing data for smoking/obesity in a fraction of participants further restricted the size of our study sample for stratified analyses and assessment of effect modification. Second, our examination of genetic variation within miRNA gene loci and miRNA-targeted mRNAs was not comprehensive. Of the ~1600 miRNAs deposited in miRBase, only ~200 were represented in our analysis. We used stringent rather than exhaustive inclusion criteria for selecting miRNA-targeted mRNAs for analysis, and prioritized targets for which expression of cognate miRNAs has been reported in esophageal tissue. While this category of variants could have been vastly expanded based on bioinformatic predictions, the majority of such predicted miRNA:mRNA interactions are not validated, and are of unknown biological significance.

In sum, our results do not provide evidence for an association between common genetic variation in components of the miRNA biogenesis core pathway and altered risk of EA or BE. Additional studies may be justified to examine potential associations between unassessed germline variants in miRNA gene loci and miRNA target genes and risk of these conditions. Validation in independent study populations, coupled with functional evaluation of the biological effects of specific SNPs, would be needed to substantiate any polymorphisms as causal risk factors for BE or EA.

## Supporting Information

S1 Fig Selection of miRNA-related SNPs. (PDF)

S2 Fig Scree plot of percent variance accounted for versus principal component index. (PDF)

S3 Fig Scatter plots of pairwise comparisons among the first four principal components. (PDF)

S4 Fig Histograms of P-values obtained from primary logistic regression analyses. (PDF)

S1 Table miRNAs reported to be expressed in normal squamous epithelium of the esophagus, BE, dysplastic epithelium, or EA. (PDF)

S2 Table MiRNA-related SNPs previously reported to be associated with cancer susceptibility. (XLSX)

S3 Table MiRNA-related SNPs selected for analysis. (XLSX)

S4 Table Three SNPs from the intersection of Tables 2 & 3, and risk of EA, BE, or [EA/BE]. (PDF)

S5 Table MiRNA-related SNPs and risk of EA stratified by smoking history or BMI. (PDF)

S6 Table MiRNA-related SNPs and risk of BE stratified by smoking history or BMI. (PDF)

S7 Table MiRNA-related SNPs and risk of EA/BE. (PDF)

S8 Table Five significant miRNA-related SNPs from Ye et al. [40] and risk of EA or BE. (PDF)
