# Supplementary figures and images for: MiRNA-Related SNPs and Risk of Esophageal Adenocarcinoma and Barrett’s Esophagus: Post Genome-Wide Association Analysis in the BEACON Consortium
Source: PLoS One. 2015 Jun 3;10(6):e0128617. doi: 10.1371/journal.pone.0128617 (PMC4454432; doi:10.1371/journal.pone.0128617)

**S1 Fig. Selection of miRNA-related SNPs.**

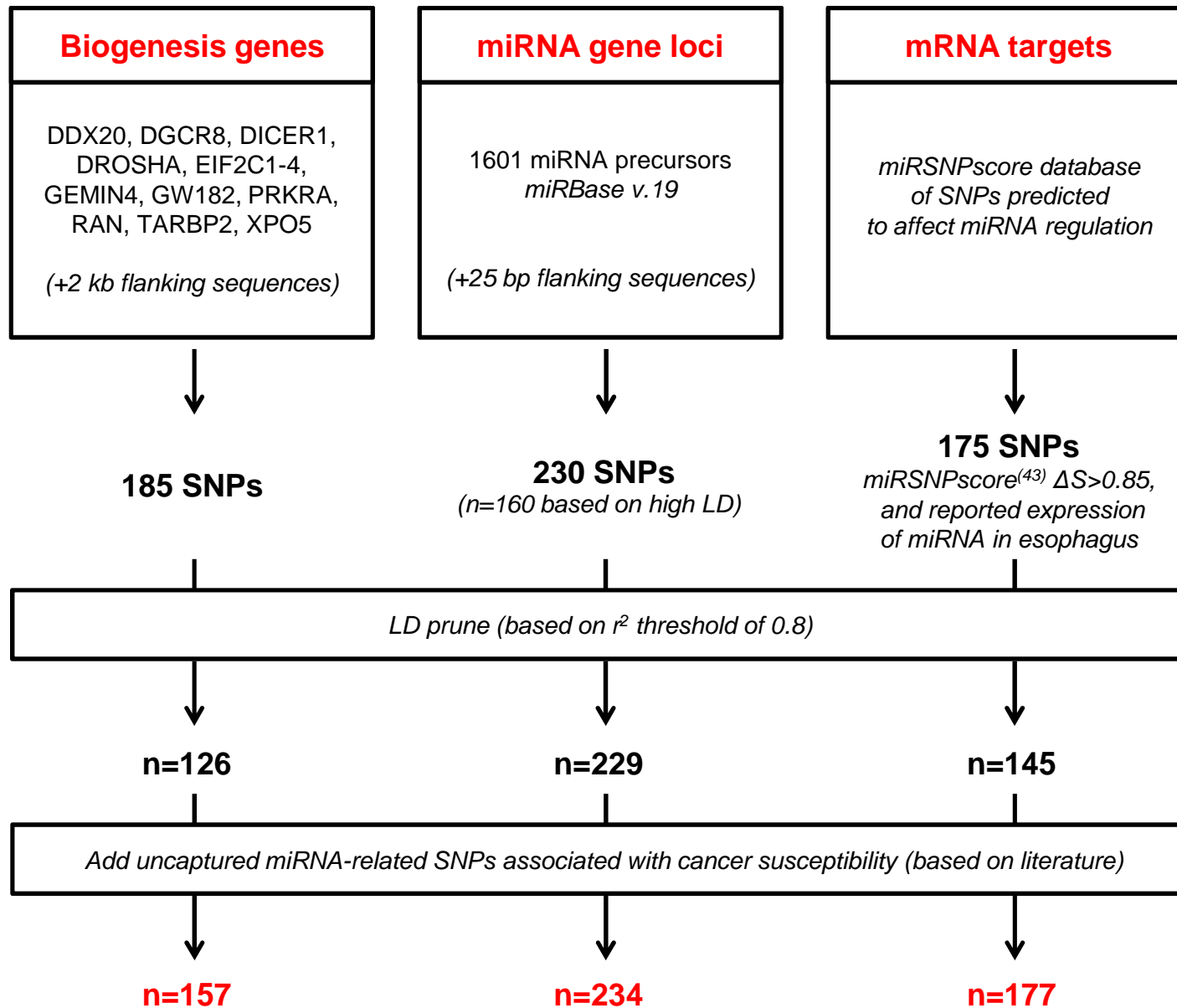

Supplement: S1 Fig — (PDF) [file pone.0128617.s001.pdf]

**S2 Fig. Scree plot of percent variance accounted for versus principal component index.**

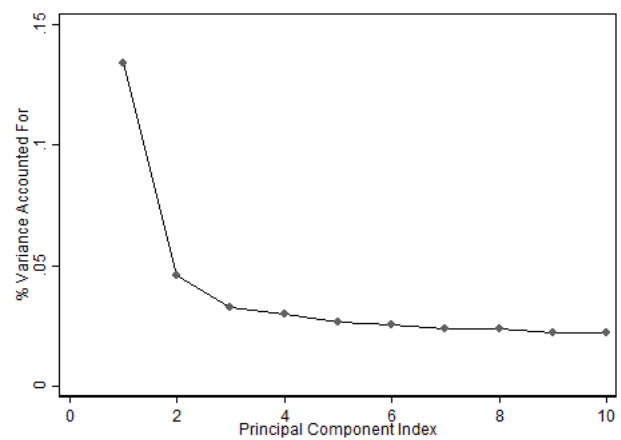

Supplement: S2 Fig — (PDF) [file pone.0128617.s002.pdf]

**S3 Fig. Scatter plots of pairwise comparisons among the first four principal components.**

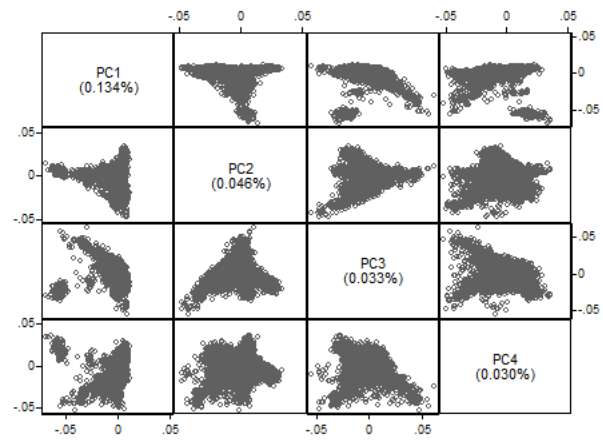

Supplement: S3 Fig — (PDF) [file pone.0128617.s003.pdf]
