# Supplementary material for: MiRNA-Related SNPs and Risk of Esophageal Adenocarcinoma and Barrett’s Esophagus: Post Genome-Wide Association Analysis in the BEACON Consortium
Source: PLoS One. 2015 Jun 3;10(6):e0128617. doi: 10.1371/journal.pone.0128617 (PMC4454432; doi:10.1371/journal.pone.0128617)

S4 Fig. Histograms of P-values obtained from logistic regression analyses assessing associations between SNPs in the indicated category and risk of EA. (A) miRNA biogenesis genes, (B) miRNA genes, (C) miRNA target genes, (D) all SNPs.

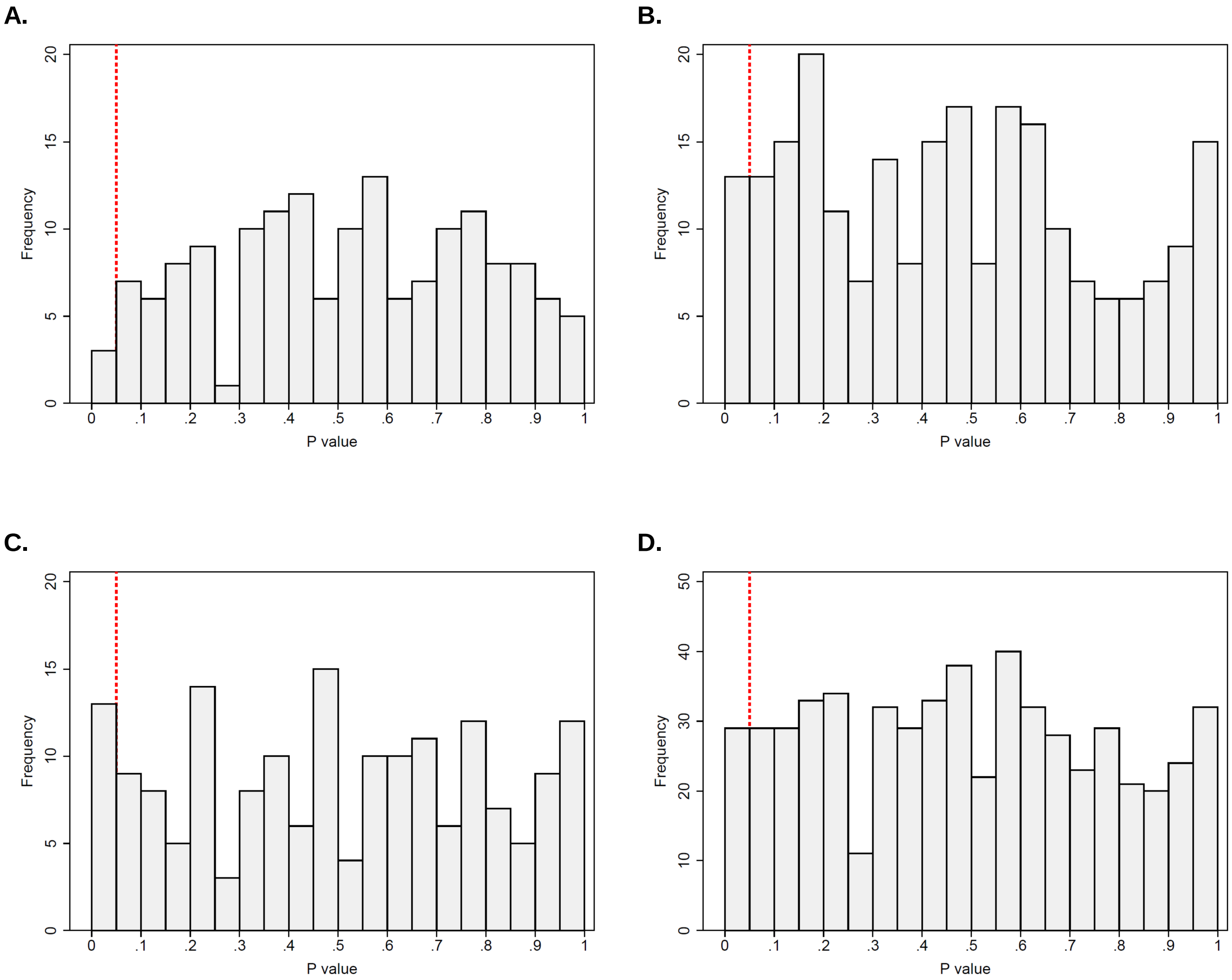

Supplement: S4 Fig — (PDF) [file pone.0128617.s004.pdf]
