# Supplementary material for: MiRNA-Related SNPs and Risk of Esophageal Adenocarcinoma and Barrett’s Esophagus: Post Genome-Wide Association Analysis in the BEACON Consortium
Source: PLoS One. 2015 Jun 3;10(6):e0128617. doi: 10.1371/journal.pone.0128617 (PMC4454432; doi:10.1371/journal.pone.0128617)
Supplement: S1 Table — (PDF) [file pone.0128617.s005.pdf]

S1 Table. miRNAs reported to be expressed in normal squamous epithelium of the esophagus, BE, dysplastic epithelium, or EA.

**1**	let-7a	**32**	miR-30c	**63**	miR-148b	**94**	miR-215	**125**	miR-519d
**2**	let-7a-1	**33**	miR-30d	**64**	miR-149	**95**	miR-216a	**126**	miR-543
**3**	let-7a-2	**34**	miR-30e	**65**	miR-152	**96**	miR-216b	**127**	miR-548b-3p
**4**	let-7b	**35**	miR-31	**66**	miR-155	**97**	miR-219-5p	**128**	miR-557
**5**	let-7c	**36**	miR-32	**67**	miR-181a	**98**	miR-221	**129**	miR-560
**6**	let-7d	**37**	miR-33a	**68**	miR-181a-1	**99**	miR-222	**130**	miR-605
**7**	let-7f	**38**	miR-33b	**69**	miR-181a-2	**100**	miR-223	**131**	miR-615-3p
**8**	let-7i	**39**	miR-92-1	**70**	miR-181b	**101**	miR-224	**132**	miR-617
**9**	miR-7	**40**	miR-92a	**71**	miR-187	**102**	miR-326	**133**	miR-630
**10**	miR-10a	**41**	miR-93	**72**	miR-190	**103**	miR-330-5p	**134**	miR-636
**11**	miR-10b	**42**	miR-99a	**73**	miR-191	**104**	miR-338-3p	**135**	miR-663
**12**	miR-15b	**43**	miR-99b	**74**	miR-192	**105**	miR-338-5p		
**13**	miR-17	**44**	miR-100	**75**	miR-193a-3p	**106**	miR-342-3p		
**14**	miR-17-5p	**45**	miR-101	**76**	miR-193a-5p	**107**	miR-342-5p		
**15**	miR-20a	**46**	miR-103	**77**	miR-193b	**108**	miR-345		
**16**	miR-20b	**47**	miR-103-1	**78**	miR-194	**109**	miR-355-5p		
**17**	miR-21	**48**	miR-106a	**79**	miR-194-1	**110**	miR-369-3p		
**18**	miR-23a	**49**	miR-107	**80**	miR-195	**111**	miR-369-5p		
**19**	miR-23b	**50**	miR-125b	**81**	miR-196a	**112**	miR-370		
**20**	miR-24	**51**	miR-126	**82**	miR-197	**113**	miR-375		
**21**	miR-25	**52**	miR-135a	**83**	miR-199a*	**114**	miR-409-3p		
**22**	miR-26b	**53**	miR-140-3p	**84**	miR-199a-3p	**115**	miR-422b		
**23**	miR-27a	**54**	miR-140-5p	**85**	miR-199a-5p	**116**	miR-424		
**24**	miR-27b	**55**	miR-143	**86**	miR-199b-3p	**117**	miR-451		
**25**	miR-28-3p	**56**	miR-144	**87**	miR-199b-5p	**118**	miR-483-3p		
**26**	miR-28-5p	**57**	miR-145	**88**	miR-200a*	**119**	miR-494		
**27**	miR-29a	**58**	miR-146a	**89**	miR-200c	**120**	miR-497		
**28**	miR-29b	**59**	miR-146b-3p	**90**	miR-203	**121**	miR-509-3p		
**29**	miR-29c	**60**	miR-146b-5p	**91**	miR-205	**122**	miR-509-5p		
**30**	miR-30a	**61**	miR-147	**92**	miR-210	**123**	miR-513		
**31**	miR-30b	**62**	miR-148a	**93**	miR-214	**124**	miR-516a-5p		
