# Supplementary material for: MiRNA-Related SNPs and Risk of Esophageal Adenocarcinoma and Barrett’s Esophagus: Post Genome-Wide Association Analysis in the BEACON Consortium
Source: PLoS One. 2015 Jun 3;10(6):e0128617. doi: 10.1371/journal.pone.0128617 (PMC4454432; doi:10.1371/journal.pone.0128617)
Supplement: S4 Table — (PDF) [file pone.0128617.s008.pdf]

S4 Table. Three SNPs from the intersection of Tables 2 & 3, and risk of EA, BE, or [EA/BE].

				EA			BE			EA/BE		
	SNP	Gene	Alleles†	OR*	95% CI	P	OR*	95% CI	P	OR*	95% CI	P
**1**	rs1644730	RDH8	A/T	0.88	(0.81-0.95)	0.0011	0.93	(0.86-1.00)	0.0384	0.91	(0.85-0.97)	0.0026
**2**	rs12534337	miR-4467	A/G	1.28	(1.06-1.55)	0.0095	1.25	(1.05-1.48)	0.0106	1.27	(1.09-1.48)	0.0027
**3**	rs7526812	miR-3117	C/T	1.14	(1.02-1.27)	0.0195	1.11	(1.00-1.22)	0.0456	1.12	(1.03-1.22)	0.0112

†Minor/major alleles, *OR adjusted for age, sex, ev1-ev4 using additive model (per-allele)
