# Supplementary material for: MiRNA-Related SNPs and Risk of Esophageal Adenocarcinoma and Barrett’s Esophagus: Post Genome-Wide Association Analysis in the BEACON Consortium
Source: PLoS One. 2015 Jun 3;10(6):e0128617. doi: 10.1371/journal.pone.0128617 (PMC4454432; doi:10.1371/journal.pone.0128617)
Supplement: S5 Table — (PDF) [file pone.0128617.s009.pdf]

S5 Table. MiRNA-related SNPs and risk of EA stratified by smoking history or BMI.

A.

	Pack-years					
	**0**	**>0 & <15**	**15-29**	**30-44**	**45+**	
**SNP**	**OR (95% CI)**<br>**P**	**OR (95% CI)**<br>**P**	**OR (95% CI)**<br>**P**	**OR (95% CI)**<br>**P**	**OR (95% CI)**<br>**P**	**P-int**
rs1644730	0.84 (0.70-1.01)<br>0.0677	0.88 (0.67-1.15)<br>0.3589	0.86 (0.65-1.13)<br>0.2678	0.91 (0.70-1.18)<br>0.4744	0.86 (0.66-1.11)<br>0.2388	0.97
rs17880825	0.97 (0.55-1.71)<br>0.9086	1.03 (0.43-2.46)<br>0.956	4.87 (1.94-12.22)<br>0.0007	1.70 (0.65-4.41)<br>0.2795	1.98 (0.68-5.79)<br>0.2116	0.10
rs1045968	0.97 (0.75-1.26)<br>0.8313	1.10 (0.77-1.58)<br>0.6062	1.10 (0.78-1.56)<br>0.5882	1.15 (0.78-1.68)<br>0.4828	1.51 (1.04-2.20)<br>0.0302	0.07
rs1050629	0.65 (0.36-1.18)<br>0.1561	2.22 (1.05-4.69)<br>0.0371	1.71 (0.75-3.94)<br>0.2047	1.41 (0.65-3.06)<br>0.3834	1.29 (0.61-2.74)<br>0.5015	0.60
rs2075993	1.02 (0.85-1.22)<br>0.8498	0.82 (0.63-1.08)<br>0.1554	0.91 (0.69-1.20)<br>0.5034	0.79 (0.59-1.05)<br>0.0982	0.82 (0.64-1.06)<br>0.1279	0.45
kgp1460594	1.23 (0.89-1.72)<br>0.208	1.83 (1.11-3.02)<br>0.0186	1.11 (0.69-1.79)<br>0.669	1.04 (0.66-1.66)<br>0.8573	1.15 (0.72-1.83)<br>0.5503	0.41

B.

	BMI				
	**<25**	**25-29.9**	**30-34.9**	**35+**	
**SNP**	**OR (95% CI)**<br>**P**	**OR (95% CI)**<br>**P**	**OR (95% CI)**<br>**P**	**OR (95% CI)**<br>**P**	**P-int**
rs1644730	0.74 (0.60-0.91)<br>0.005	0.89 (0.76-1.05)<br>0.1542	0.86 (0.66-1.12)<br>0.2632	0.87 (0.58-1.32)<br>0.5253	0.21
rs17880825	1.02 (0.52-1.99)<br>0.9619	1.66 (0.93-2.94)<br>0.0837	1.32 (0.55-3.17)<br>0.5356	0.71 (0.16-3.15)<br>0.6564	0.92
rs1045968	1.17 (0.88-1.54)<br>0.2813	1.08 (0.87-1.35)<br>0.488	1.06 (0.74-1.50)<br>0.7591	1.76 (0.97-3.22)<br>0.065	0.39
rs1050629	1.53 (0.81-2.92)<br>0.1917	1.28 (0.83-1.96)<br>0.2606	0.92 (0.42-2.05)<br>0.8419	1.37 (0.31-6.05)<br>0.6821	0.97
rs2075993	0.96 (0.78-1.18)<br>0.6851	0.80 (0.68-0.94)<br>0.0071	0.87 (0.67-1.13)<br>0.2869	0.93 (0.63-1.39)<br>0.7341	0.34
kgp1460594	1.65 (1.15-2.36)<br>0.0062	1.40 (1.04-1.87)<br>0.0259	0.91 (0.60-1.37)<br>0.6389	0.88 (0.42-1.85)<br>0.7291	0.09

*OR adjusted for age, sex, ev1-ev4, using additive model (per-allele), #P-value for coefficient of product term included in the logistic model
