# Supplementary material for: MiRNA-Related SNPs and Risk of Esophageal Adenocarcinoma and Barrett’s Esophagus: Post Genome-Wide Association Analysis in the BEACON Consortium
Source: PLoS One. 2015 Jun 3;10(6):e0128617. doi: 10.1371/journal.pone.0128617 (PMC4454432; doi:10.1371/journal.pone.0128617)
Supplement: S6 Table — (PDF) [file pone.0128617.s010.pdf]

S6 Table. MiRNA-related SNPs and risk of BE stratified by smoking history or BMI.

A.

	Pack-years					
	**0**	**>0 & <15**	**15-29**	**30-44**	**45+**	
**SNP**	**OR (95% CI)**<br>**P**	**OR (95% CI)**<br>**P**	**OR (95% CI)**<br>**P**	**OR (95% CI)**<br>**P**	**OR (95% CI)**<br>**P**	**P-int**
rs3785722	0.87 (0.76-0.99)<br>0.0391	0.87 (0.69-1.09)<br>0.22	0.96 (0.75-1.24)<br>0.7665	0.77 (0.58-1.02)<br>0.0681	0.94 (0.73-1.20)<br>0.616	0.39
rs8192593	0.56 (0.39-0.81)<br>0.0023	0.66 (0.37-1.18)<br>0.161	1.73 (0.92-3.25)<br>0.0876	0.89 (0.42-1.90)<br>0.7621	1.01 (0.54-1.89)<br>0.9829	0.25
rs1043681	0.89 (0.77-1.03)<br>0.1072	0.89 (0.71-1.14)<br>0.3603	0.87 (0.67-1.14)<br>0.3091	0.71 (0.51-0.97)<br>0.0308	1.04 (0.80-1.35)<br>0.7921	0.82
rs10906086	1.07 (0.93-1.23)<br>0.3616	1.16 (0.93-1.43)<br>0.186	1.06 (0.83-1.35)<br>0.6517	1.47 (1.11-1.94)<br>0.0066	0.90 (0.70-1.16)<br>0.4166	0.34

B.

	BMI				
	**<25**	**25-29.9**	**30-34.9**	**35+**	
**SNP**	**OR (95% CI)**<br>**P**	**OR (95% CI)**<br>**P**	**OR (95% CI)**<br>**P**	**OR (95% CI)**<br>**P**	**P-int**
rs3785722	0.87 (0.73-1.03)<br>0.1078	0.90 (0.78-1.03)<br>0.1122	0.80 (0.65-0.99)<br>0.0399	0.94 (0.69-1.27)<br>0.6744	0.47
rs8192593	0.78 (0.49-1.24)<br>0.3014	0.78 (0.55-1.12)<br>0.179	0.50 (0.29-0.86)<br>0.0122	1.07 (0.45-2.52)<br>0.879	0.63
rs1043681	0.89 (0.74-1.07)<br>0.2142	0.82 (0.71-0.95)<br>0.0064	1.02 (0.81-1.28)<br>0.8652	1.21 (0.86-1.70)<br>0.267	0.08
rs10906086	1.26 (1.06-1.49)<br>0.0088	0.97 (0.84-1.10)<br>0.6096	1.08 (0.87-1.34)<br>0.485	1.09 (0.80-1.47)<br>0.5923	0.06

*OR adjusted for age, sex, ev1-ev4, using additive model (per-allele), #P-value for coefficient of product term included in the logistic model
