# Supplementary material for: MiRNA-Related SNPs and Risk of Esophageal Adenocarcinoma and Barrett’s Esophagus: Post Genome-Wide Association Analysis in the BEACON Consortium
Source: PLoS One. 2015 Jun 3;10(6):e0128617. doi: 10.1371/journal.pone.0128617 (PMC4454432; doi:10.1371/journal.pone.0128617)
Supplement: S7 Table — (PDF) [file pone.0128617.s011.pdf]

S7 Table. MiRNA-related SNPs and risk of EA/BE.

	Category	SNP	Gene	Alleles†	Controls N	Controls MAF‡	EA/BE cases N	EA/BE cases MAF‡	OR*	95% CI	P	q
**1**	Target	rs1644730	RDH8	A/T	3200	0.473	5776	0.448	0.91	(0.85-0.97)	0.0026	0.75
**2**	miRNA	rs12534337	miR-4467	A/G	3203	0.042	5778	0.053	1.27	(1.09-1.48)	0.0027	0.75
**3**	miRNA	rs3785722	miR-1269b	A/G	3204	0.449	5779	0.428	0.91	(0.86-0.97)	0.0049	0.80
**4**	miRNA	rs7000768	miR-3686	G/A	3205	0.304	5779	0.323	1.09	(1.02-1.17)	0.0104	0.80
**5**	miRNA	rs10906086	miR-548ak	C/A	3204	0.469	5776	0.487	1.09	(1.02-1.16)	0.0106	0.80
**6**	miRNA	rs7526812	miR-3117	C/T	3205	0.151	5778	0.162	1.12	(1.03-1.22)	0.0112	0.80
**7**	Biogenesis	rs8192593	TARBP2	T/C	3202	0.040	5776	0.035	0.81	(0.69-0.96)	0.0123	0.80
**8**	miRNA	rs2114358	miR-1206	G/A	3205	0.390	5779	0.409	1.08	(1.02-1.16)	0.0139	0.80
**9**	Target	rs1043681	THAP3	C/T	3205	0.316	5779	0.304	0.92	(0.86-0.98)	0.0153	0.80
**10**	Target	rs1050629	MLF2	A/G	3204	0.025	5779	0.031	1.27	(1.04-1.55)	0.0166	0.80
**11**	Target	rs1045968	PRRT2	A/C	3204	0.141	5778	0.154	1.12	(1.02-1.22)	0.0167	0.80
**12**	miRNA	rs12564376	miR-4421	A/G	3205	0.039	5779	0.032	0.81	(0.69-0.97)	0.0184	0.80
**13**	Target	rs2075993	E2F2	T/C	3204	0.499	5778	0.483	0.93	(0.87-0.99)	0.0197	0.80
**14**	Biogenesis	rs12044203	EIF2C4	A/G	3195	0.021	5772	0.014	0.75	(0.58-0.96)	0.0209	0.80
**15**	Biogenesis	rs4351606	EIF2C3	A/G	3203	0.057	5776	0.048	0.85	(0.74-0.98)	0.0242	0.80
**16**	Target	rs1043641	ACBD3	A/G	3205	0.172	5779	0.159	0.91	(0.83-0.99)	0.0267	0.80
**17**	Biogenesis	rs2944760	EIF2C2	C/A	3199	0.199	5778	0.189	0.91	(0.84-0.99)	0.0272	0.80
**18**	Target	rs11550670	STXBP6	T/C	3202	0.204	5771	0.219	1.09	(1.01-1.18)	0.0278	0.80
**19**	miRNA	rs3787547	miR-4756	A/G	3203	0.435	5779	0.416	0.93	(0.87-0.99)	0.0281	0.80
**20**	Biogenesis	rs11247946	LIN28	G/A	3205	0.345	5778	0.329	0.93	(0.87-0.99)	0.0312	0.80
**21**	miRNA	rs653667	miR-4632	G/T	3177	0.367	5700	0.352	0.93	(0.87-0.99)	0.0315	0.80
**22**	Biogenesis	kgp1460594	XPO5	A/G	3205	0.075	5776	0.086	1.14	(1.01-1.28)	0.0318	0.80
**23**	Biogenesis	rs595055	EIF2C1	C/T	3203	0.142	5778	0.127	0.90	(0.82-0.99)	0.0342	0.80
**24**	Target	rs11169571	ATF1	C/T	3204	0.391	5778	0.415	1.07	(1.00-1.14)	0.0357	0.80
**25**	Target	rs9804386	MORN4	C/T	3205	0.200	5779	0.217	1.09	(1.00-1.18)	0.0375	0.80
**26**	miRNA	rs10862193	miR-617	G/A	3201	0.428	5769	0.415	0.93	(0.88-1.00)	0.0387	0.80
**27**	Target	rs8044745	NECAB2	T/C	3205	0.050	5777	0.043	0.85	(0.73-0.99)	0.0395	0.80
**28**	Target	rs17465826	SF3A3	G/T	3204	0.041	5774	0.045	1.18	(1.01-1.39)	0.0415	0.80
**29**	Target	rs1367	SCUBE2	G/A	3205	0.075	5777	0.066	0.88	(0.78-1.00)	0.0441	0.80
**30**	Biogenesis	rs7702984	DROSHA	G/A	3204	0.012	5778	0.009	0.73	(0.54-1.00)	0.0466	0.80
**31**	miRNA	rs12461701	miR-3188	A/G	3204	0.278	5776	0.263	0.93	(0.87-1.00)	0.0477	0.80

†Minor/major alleles, ‡Minor allele frequency, *OR adjusted for age, sex, ev1-ev4, using additive model (per-allele). Variants highlighted in yellow were nominally significant in both the EA and BE analysis.
