# Supplementary material for: MiRNA-Related SNPs and Risk of Esophageal Adenocarcinoma and Barrett’s Esophagus: Post Genome-Wide Association Analysis in the BEACON Consortium
Source: PLoS One. 2015 Jun 3;10(6):e0128617. doi: 10.1371/journal.pone.0128617 (PMC4454432; doi:10.1371/journal.pone.0128617)
Supplement: S8 Table — (PDF) [file pone.0128617.s012.pdf]

S8 Table. Five significant miRNA-related SNPs from Ye et al. (40) and risk of EA (A) or BE (B).

A.

	SNP	Gene	Alleles†	Controls N	Controls MAF‡	EA cases N	EA cases MAF‡	OR*	95% CI	P
**1**	rs213210	pri-miR-219-1	G/A	3204	0.069	2491	0.080	1.12	(0.96-1.30)	0.147
**2**	rs6505162	miR-423, -3184	C/A	3207	0.455	2495	0.455	1.04	(0.96-1.12)	0.348
**3**	rs14035	RAN	A/G	3206	0.310	2494	0.316	1.04	(0.95-1.13)	0.398
**4**	rs11614913	miR-196a-2	T/C	3204	0.402	2495	0.416	1.02	(0.94-1.10)	0.629
**5**	rs11077	XPO5	C/A	3206	0.415	2495	0.423	1.01	(0.93-1.10)	0.749

B.

	SNP	Gene	Alleles†	Controls N	Controls MAF‡	BE cases N	BE cases MAF‡	OR*	95% CI	P
**1**	rs213210	pri-miR-219-1	G/A	3201	0.069	3284	0.074	1.03	(0.90-1.19)	0.623
**2**	rs6505162	miR-423, -3184	C/A	3204	0.455	3285	0.449	1.01	(0.94-1.08)	0.831
**3**	rs14035	RAN	A/G	3203	0.310	3288	0.304	0.99	(0.92-1.07)	0.785
**4**	rs11614913	miR-196a-2	T/C	3201	0.402	3285	0.410	1.00	(0.93-1.07)	0.993
**5**	rs11077	XPO5	C/A	3203	0.415	3287	0.427	1.04	(0.97-1.12)	0.305

†Minor/major alleles, ‡Minor allele frequency, *OR adjusted for age, sex, ev1-ev4, using additive model (per-allele)
